# CEGO: C++11 Evolutionary Global Optimization

**Published:** 2018

**Authors:** Ian H. Bell

**Affiliations:** 1 National Institute of Standards and Technology, Boulder, CO, USA

## Summary

Global optimization is an algorithmic need that is ubiquitous throughout the natural sciences, engineering, and other technical spheres. It is a non-trivial task, particularly when the function to be optimized has many local minima and the optimization algorithm may get trapped in the local minima of the function to be optimized. For that reason, many competing approaches have been proposed for global optimization, especially those inspired by nature. The goal of the library proposed here is to develop a user-friendly framework in C++11 (with wrappers for Python) that can be used to successfully and efficiently carry out global optimization of challenging cost functions with minimum expertise required.

CEGO (C++11 Evolutionary Global Optimization) is a C++11-based optimization library that minimizes an arbitrary cost function. In C++, the cost function to be minimized is of type std::function<double(const CEGO::AbstractIndividual *)>, where CEGO::AbstractIndividual is the base class for an individual in the population of candidate solutions. The independent variables to be optimized are of type std::vector<CEGO::numberish>, where the datatype CEGO::numberish can accept both discrete (integer) and continuous values.

A brief summary of the functionality of CEGO includes:

- The implementation of the ALPS algorithm (Hornby, 2006, 2009) for age-layering several optimization runs together. The layers interface is based on migration of younger individuals in the population into older layers. If the individual is too old, and does not dominate another individual in its next layer, it is removed from the population. Age layering can be disabled through the use of a single layer if desired.
- Latin hypercube sampling to generate the initial population of individuals in the population.
- A generic architecture for evolving the layered population(s). In the current version, differential evolution (Storn & Price, 1997) is the default evolving method, though an extensible API is available that allows for plug-and-play of alternative population evolution methods. Flags for the evolver are handled in a generic way with a JavaScript Object Notation (JSON) structure.
- Use of native C++11 threads (with a thread pool) to parallelize the evaluation of the cost function, allowing for a nearly-linear speedup as more computational cores are made available.
- Ability to log all inputs and outputs (along with an optional filtering function) for further analysis of the progress of the optimization.
- A single-threaded Python wrapper (PyCEGO) is written with pybind11^1^ and is used to demonstrate the functionality of the library, though it cannot fully leverage the parallelism available in CEGO at the C++ level.

A few Jupyter notebooks (Kluyver et al., 2016; Pérez & Granger, 2007) are provided as examples that implement:

- optimization of cost functions of two- and ten-dimensional continuous variables.
- the mixed-integer nonlinear optimization problems of the constrained optimization of a pressure vessel mass and dimensionally-constrained spring (Sandgren, 1990)
- inverse modeling of Gaussian bumps.

All global optimization problems successfully obtain the minimum value from the literature, or better. Furthermore, a binder (Jupyter et al., 2018) environment has been configured such that the Jupyter notebooks can be run interactively in an internet browser without any installation on the user’s computer.

An example is given here of the global optimization of the modified hundred-digit optimization problem (Townsend, 2014, Eq. 5.15), a function with 9,318 different local minima in [−1,1]×[−1,1]. CEGO finds the correct global minimum value of −3.398166873463248.


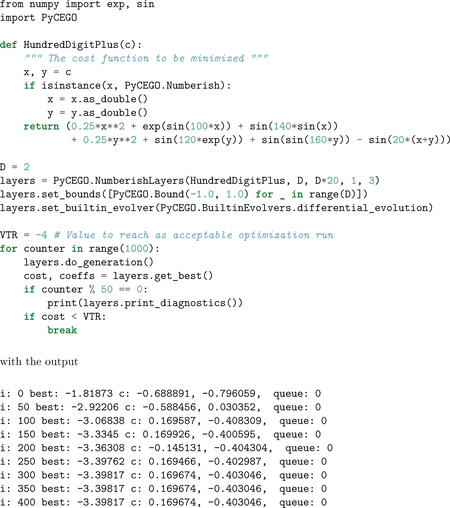



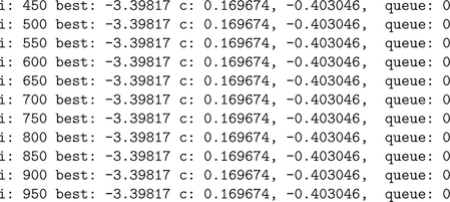


## Footnotes

1 https://github.com/pybind/pybind11
